# Colonic Gangrene: A Sequela of Coronavirus Disease 2019

**DOI:** 10.7759/cureus.14687

**Published:** 2021-04-26

**Authors:** Rahul Varshney, Nalini Bansal, Archana Khanduri, Jyoti Gupta, Rahul Gupta

**Affiliations:** 1 Anesthesia and Critical Care, Synergy Institute of Medical Sciences, Dehradun, IND; 2 Department of Histopathology, SRL Limited, Fortis Escorts Heart Institute, New Delhi, IND; 3 Gastrointestinal Surgery, Synergy Institute of Medical Sciences, Dehradun, IND; 4 Radiation Oncology, Himalayan Institute of Medical Sciences, Dehradun, IND

**Keywords:** sigmoid colon, covid-19, small vessel vasculitis, intestinal ischemia, colectomy, sars-cov-2, bowel gangrene

## Abstract

Initially considered to be a respiratory disease, coronavirus disease 2019 (COVID-19) is now recognized as a multisystem disease known to affect all the major organs, including the gastrointestinal system. Based on recent studies, severe acute respiratory syndrome coronavirus 2 causes dysregulation of multiple biological pathways, triggers an exaggerated immune response, and affects multiple organs. The gastrointestinal symptoms in COVID-19 are common but often overlooked. We report the case of a 50-year-old female with a recent history of COVID-19 presenting with complaints of abdominal pain and constipation. Initially, the patient was treated for respiratory symptoms and discharged home. Subsequently, she was re-admitted and diagnosed with colonic obstruction on radiology. Laparotomy revealed descending and sigmoid colonic gangrene requiring left colectomy. This case highlights the uncommon but severe gastrointestinal manifestations of COVID-19.

## Introduction

Coronavirus disease 2019 (COVID-19) is a multisystem disease caused by severe acute respiratory syndrome coronavirus 2 (SARS-CoV-2). Since its first description in Wuhan, China, COVID-19 has spread rapidly across the world and has become one of the most lethal pandemics known to humans. It is clinically suspected in patients with the typical symptoms of fever, cough, anosmia, breathlessness, fatigue, and headache [1]. However, up to 15% of cases can develop gastrointestinal symptoms such as nausea, vomiting, anorexia, diarrhea, and abdominal pain [2]. These gastrointestinal manifestations of COVID-19 are most often self-limiting. In about 10% of cases, gastrointestinal symptoms can be present without respiratory manifestations [2]. Hence, a high index of suspicion is required for timely diagnosis of COVID-19. Additionally, COVID-19 is associated with coagulopathy and vasculitis, which correlate with the disease severity and increase the risk of mortality [3-7]. Involvement of the mesenteric vessels in COVID-19 patients can predispose to the development of intestinal ischemia [8-11]. Due to predominant respiratory manifestations, the gastrointestinal manifestations of COVID-19 are often overlooked, leading to delayed diagnosis. We report a case of acute colonic gangrene involving descending and sigmoid colon in a female patient after two weeks of diagnosing SAR-CoV-2 infection.

## Case presentation

**Figure 1:**
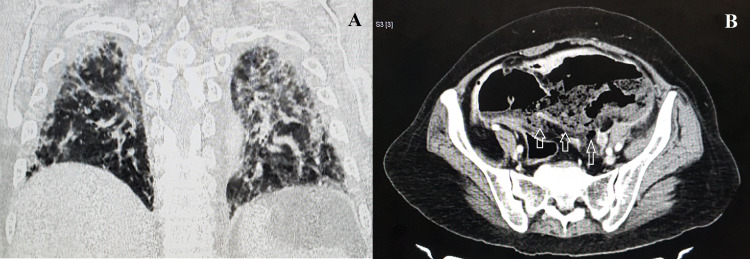
CT of the chest showing centrilobular ground glass opacities involving both the lungs and suggestive of COVID-19 pneumonia (A) and grossly dilated sigmoid colon with imperceptible posterior wall (arrows) (B). CT: computed tomography; COVID-19: coronavirus disease 2019

A 50-year-old hypertensive female presented with abdominal pain and constipation for five days. She was diagnosed with COVID-19 and admitted to another hospital for two weeks due to respiratory symptoms. She was discharged five days before presenting with abdominal symptoms. On clinical examination, the patient had a tender hypogastric lump. Rectal examination showed normal-colored stools. Blood investigations showed leucocytosis. COVID-19 reverse transcriptase-polymerase chain reaction was negative at the time of admission. Computed tomography (CT) of the chest revealed centrilobular ground-glass opacities involving both the lungs suggestive of COVID-19 pneumonia (Figure 1A). Contrast-enhanced CT of the abdomen and pelvis revealed grossly dilated distal segment of the descending colon and sigmoid colon with multiple diverticulosis. The posterior wall of the sigmoid colon was imperceptible possibly due to the ruptured diverticulum (Figure 1B).

**Figure 2:**
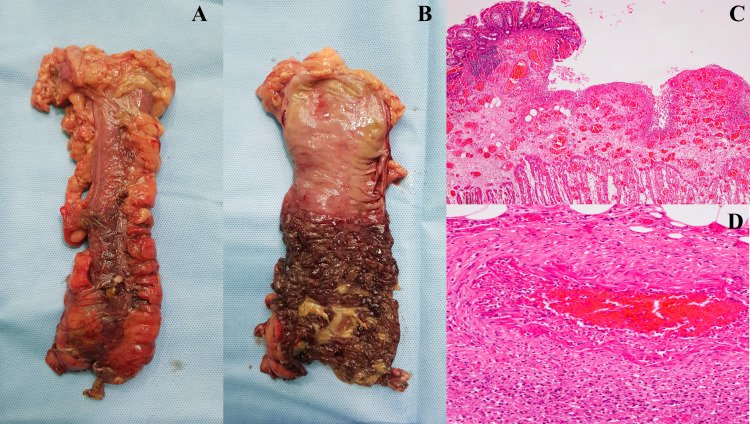
The macroscopic appearance of the descending colon from the serosal side (A) and the mucosal side (B) showing a distinct line of demarcation between healthy and ischemic bowel segments. Microscopic examination of the resected colon revealed denuded and sloughed mucosal surface (H&E, ×10) (C). The medium-sized arteries in the mesocolon showed inflammatory changes in their walls with foci of necrosis suggestive of vasculitis (H&E, ×40) (D). H&E: hematoxylin and eosin

On laparotomy, an 8 × 10 × 8 cm feculent collection was present in the hypogastrium. The whole sigmoid colon was gangrenous. The descending colon was ischemic with the presence of multiple perforations (Figure 2). Drainage of the collection, left colectomy with transverse colostomy, and rectal stump closure (Hartmann procedure) were performed. The surgery lasted for 180 minutes with a blood loss of 200 mL. Postoperatively, the patient was shifted to the intensive care unit for mechanical ventilation and minimal vasopressor support. The patient responded to treatment and was extubated 12 hours post-surgery. The patient was started on anticoagulation (enoxaparin, 60 mg twice a day). On the third postoperative day, she developed bluish discoloration of the toes in the left foot, suggestive of impending gangrene. Subsequently, the patient developed respiratory distress and drowsiness, requiring re-intubation and mechanical ventilation. Her condition rapidly deteriorated, and she expired on the same day.

Histopathology of the resected specimen revealed denudation of the mucosal surface epithelium with loss of crypts (Figure 2C). Lamina propria appeared pink and edematous, while other areas displayed regenerating immature crypts. The medium-sized arteries contained mixed cellular inflammation and foci of necrosis (Figure 2D). Other vessels showed thickened intima with a compromised lumen (Figure 2D). These features suggested acute intestinal ischemia at different stages of development.

## Discussion

Although the thrombotic complications of COVID-19 have been widely reported, the true incidence is not known. However, due to the prominence of the pulmonary manifestations, extrapulmonary symptoms are often overlooked, resulting in a delayed diagnosis of gastrointestinal complications of COVID-19 [12]. The possible explanations for the spectrum of bowel findings in patients with COVID-19 include direct viral infection, small vessel thrombosis, or nonocclusive mesenteric ischemia [3,4]. Angiotensin-converting enzyme-2 surface expression is most abundant in the lung alveolar epithelium, enterocytes of the small intestine, and vascular endothelium, suggesting that the small bowel and vasculature may be susceptible to SARS-CoV-2 infection. Findings suggestive of SARS-CoV-2 infection having a direct inflammatory effect on the vascular endothelium have been reported [3].

Furthermore, systemic coagulopathy is common in critically ill patients with COVID-19. This coagulopathy has been proposed to be due to complement-mediated microvascular injury, immune dysregulation, anti-phospholipid syndrome-like state, and vascular imaging abnormalities [4,5]. In COVID-19-associated intestinal ischemia, bowel gangrene has been reported to occur with or without the involvement of the major arteries such as superior mesenteric artery or veins such as superior mesenteric vein and portal vein [8-11]. In the index case, the large-sized arteries were patent with no apparent atherosclerotic disease. The histopathology found mixed cellular inflammation of the medium-sized vessels with thickened intima suggestive of vasculitis. Recent studies have found that SARS-CoV-2 directly infects the endothelial cells leading to cell death and apoptosis [6]. Endothelial damage leads to microcirculatory disturbances, vasculitis, and predisposes to thrombus formation in the pulmonary vasculature and other vascular beds [3-6]. Moreover, the presence of systemic coagulopathy along with endothelial damage predisposes to the development of thromboembolic complications in COVID-19.

Treatment of COVID-19-associated intestinal ischemia is similar to that of bowel ischemia due to other causes such as atherosclerotic disease. Surgical resection of the diseased bowel along with systemic anticoagulation remains the mainstay of treatment. However, the mortality of COVID-19-related bowel ischemia is high due to the multisystem involvement.

## Conclusions

Bowel gangrene should be included in the differential diagnosis while treating COVID-19 patients with gastrointestinal symptoms. Contrast-enhanced CT of the abdomen can help make a rapid diagnosis of bowel ischemia, allow early treatment, and reduce mortality in these critically ill patients. Early initiation of anticoagulation may prevent these ischemic complications of COVID-19.
